# The Assessment of Baseline Comprehensive Geriatric Assessment Parameters in Geriatric Patients With Varying Severity of Hyponatremia at a Tertiary Care Center

**DOI:** 10.7759/cureus.21516

**Published:** 2022-01-23

**Authors:** Mayank Kapoor, Minakshi Dhar, Monika Pathania

**Affiliations:** 1 Internal Medicine, All India Institute of Medical Sciences, Rishikesh, Rishikesh, IND; 2 General Medicine, All India Institute of Medical Sciences, Rishikesh, Rishikesh, IND

**Keywords:** hindi mental state examination, hmse, frailty, timed up and go test, cognition, barthel’s index of activities of daily living, hyponatremia

## Abstract

Background and objective

The prevalence of hyponatremia is estimated to be significantly higher in the geriatric age group compared to non-geriatric patients. The clinical symptoms of hyponatremia are often subtle and interpreted as age-related in geriatric patients. In this study, we aimed to perform the baseline comprehensive geriatric assessment (CGA) among a group of geriatric population with hyponatremia.

Methods

We utilized four simple CGA parameters: the Hindi Mental State Examination (HMSE) to assess the cognition, the Barthel Index for Activities of Daily Living (ADL) for assessing the level of independence, the Timed Up and Go (TUG) test for risk of fall evaluation, and handgrip (HG) strength by hand dynamometer for frailty. All CGA parameters were analyzed at admission among 100 geriatric patients (>60 years old), and an assessment of their relationship with the severity of hyponatremia was done. An equal number of age-, comorbidity-, and reason for acute presentation-matched hyponatremic patients were enrolled as controls. The student’s t-test and analysis of variance (ANOVA) were used for evaluation. Ethical clearance was obtained from the Institutional Ethics Committee, All India Institute of Medical Sciences, Rishikesh, and informed consent from patients or next of kin was taken before enrollment.

Results

The mean age of the study population was 68.1 ± 5.8 years, with a male-to-female ratio of 3:1. All CGA parameters tested showed lower values among hyponatremic patients compared to normonatremic patients, although only ADL (71.6 ± 12.3 vs. 76.7 ± 11.5, p=0.001) and HMSE (23.4 ± 3.1 vs. 24.4 ± 2.4, p=0.01) were statistically significant. All parameters were found to be worse in the severe group compared to moderate and mild groups, but significance was found only for TUG (17.9 ± 3.4 vs. 16.4 ± 4.2 vs. 14.6 ± 3.5, p=0.003, with higher values indicating worse status) and HMSE (21.1 ± 4.0 vs. 22.6 ± 2.8 vs. 24.1 ± 2.5, p=0.0007).

Conclusion

Based on our findings, hyponatremic patients have poor baseline CGA parameter values, and the severity of hyponatremia correlates with poor motor and cognitive functions. Hence, the prompt recognition and correction of hyponatremia should be prioritized in the elderly as both these parameters significantly impact the quality of life in this population. As the severity of hyponatremia increases, the elderly tend to have a higher incidence of the two main geriatric giants: impaired cognition and falls.

## Introduction

Hyponatremia is defined as a serum sodium concentration in the blood of less than 135 mmol/L [1]. It is the most common electrolyte abnormality encountered in clinical practice, seen in 15-30% of hospitalized patients [2]. The main functions of sodium in the human body include blood volume maintenance, water balance, cell membrane potential, acid-base balance, and nerve conduction [3]. Patients may develop neurological symptoms resulting from cerebral edema induced by water movement into the brain. These include seizures, impaired mental status, coma, and even death. The prevalence of hyponatremia is estimated to be about 22.2% in the geriatric age group, while it is about 6% among non-geriatric patients. Clinical symptoms of hyponatremia are often subtle and interpreted as age-related in geriatric patients. Hyponatremia in the elderly can have a variety of presentations, including frequent falls and gait disturbances [4]. A significant association has been reported between hyponatremia and osteoporosis, falls, fractures, delirious states, cognitive impairment, dementia, and mortality [5-7]. These neurologic impairments are associated with a decreased quality of life and may even prove to be a significant cause of mortality. However, since underlying diseases such as adrenal insufficiency, heart failure, liver cirrhosis, and cancer may also affect brain function, the contribution of hyponatremia itself to neurologic manifestations remains unknown. Hence, we utilized four simple and commonly used comprehensive geriatric assessment (CGA) parameters in a real-world setting - the Hindi Mental State Examination (HMSE), the Barthel Index for Activities of Daily Living (ADL), the Timed Up and Go (TUG) test, and handgrip strength (HG) by hand dynamometer - to analyze the baseline CGA parameters with respect to the severity of hyponatremia in geriatric patients.

## Materials and methods

The study was conducted over a period of 18 months, from April 2020 to October 2021, at a tertiary care center in north India. To the best of our knowledge, no study of a similar design had been conducted in this region. Given the exploratory nature of this study, 100 study subjects were recruited as subjects; 100 consecutive patients ≥60 years of age and admitted to the geriatric unit of the Department of Internal Medicine with serum sodium <135 meq/L at the time of admission were included. An equal number of normonatremic patients who were matched for age, comorbidity, and reason for presentations were also included. Patients presenting with other recognizable causes of cognitive and motor impairment like a previous stroke, patients with head trauma, Parkinson’s disease, Alzheimer’s dementia, meningitis, encephalopathies (metabolic or other causes), those with known muscular dystrophies, spinal cord injuries, or patients in whom CGA parameters could not be assessed were excluded.

Descriptive statistics were provided for categorical variables as frequencies and were compared using the Chi-square test. Comparisons of numerical data were conducted using the t-test. All results were presented as mean ± standard deviation (SD). Findings were statistically analyzed by using Microsoft Excel (Microsoft Corporation, Redmond, WA).

Study tools used

**Figure 1:**
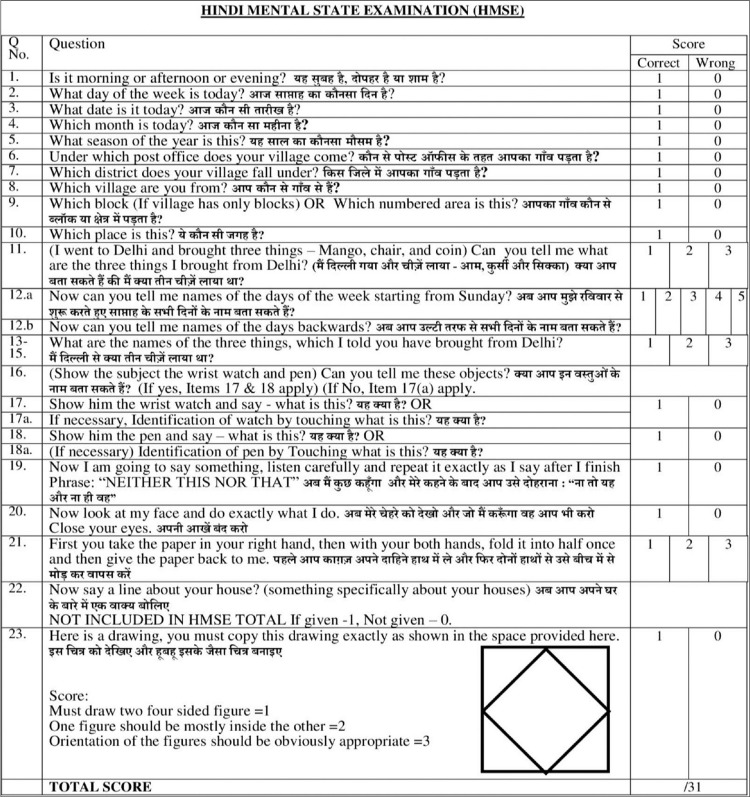
Hindi Mental State Examination

**Figure 2:**
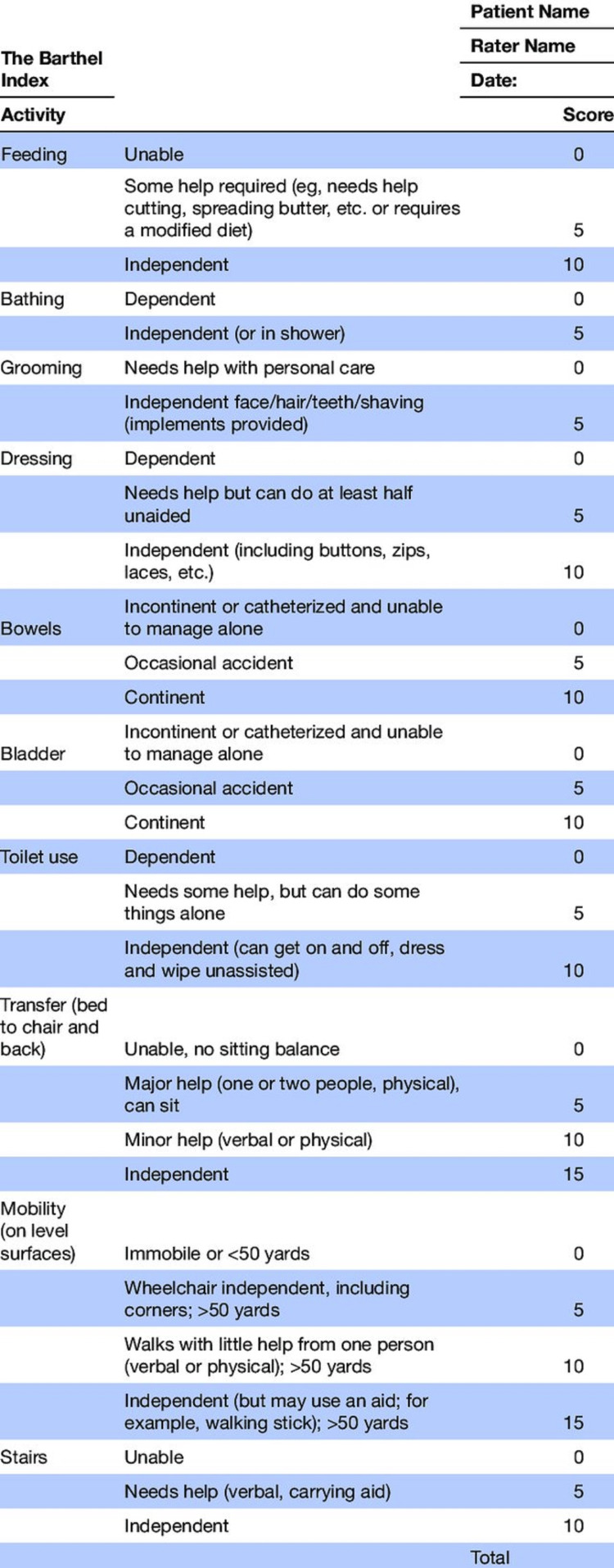
Barthel's Index for Activities of Daily Living

**Figure 3:**
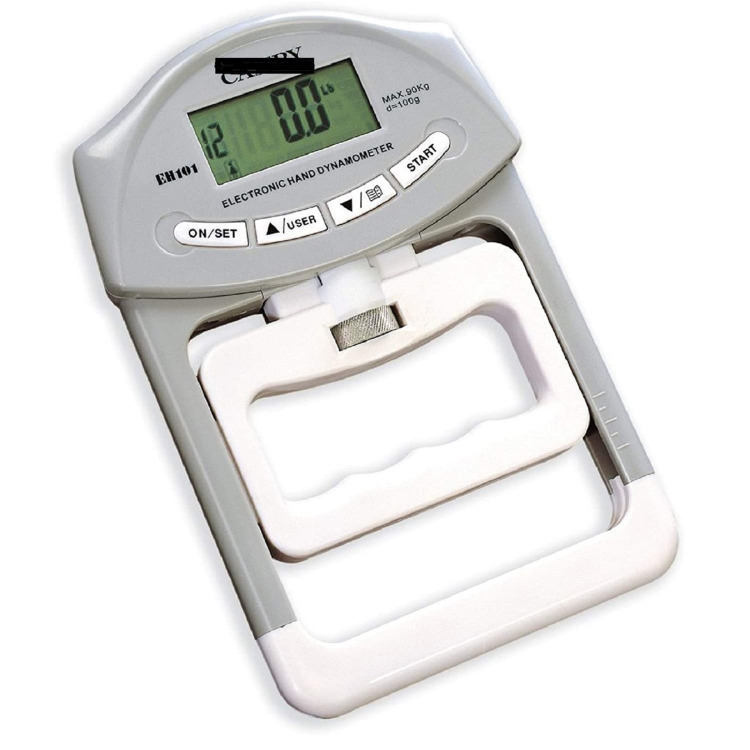
Hand dynamometre

**Figure 4:**
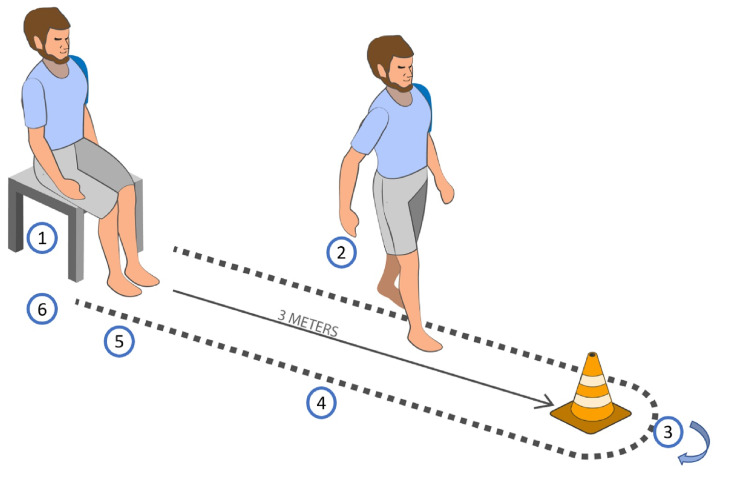
Timed Up and Go test

The cognitive abilities were assessed using the HMSE (Figure 1, Appendix). It had been developed by the Indo-U.S. Cross-National Epidemiology Study as a modified version of the Mini-Mental State Examination (MMSE), to cater to the largely rural and illiterate elderly population in India. The cut-off score for possible cognitive impairment on HMSE is 19 or below [8]. We utilized the Barthel Index for ADL (Figure 2, Appendix) and HG assessment (Figure 3, Appendix) for assessing the motor function. Mahoney and Barthel introduced the Barthel Index (called the Maryland Disability Index initially) in 1955 [9]. A score of 0-20 indicates “total” dependency, 21-60 indicates “severe” dependency, 61-90 indicates “moderate” dependency and 91-99 indicates “slight” dependency. Most studies apply the 60/61 cut-off point. HG detection was done by using a hand dynamometer [10]. The participants sit upright on a height-adjustable chair with their feet supported. The cut-off values used for HG in male and female elderly healthy populations were 28.6 and 16.4 kg, respectively [11]. The TUG test (Figure 4, Appendix) was utilized for assessing mobility. The patient was asked to get up from a chair, walk 3 meters, turn, come back and sit back in the chair. A score of ≥30 seconds suggests that the person may be prone to falls. TUG's normal value is below 12 seconds [12].

## Results

A total of 150 elderly patients with hyponatremia (serum Na+: <135 meq/L) were screened at admission during the study duration. Forty-two participants were excluded as per exclusion criteria, four patients left the hospital on request, and four died within 24 hours of admission. Finally, a total of 100 hyponatremia patients and an equal number of normonatremic patients were enrolled for analysis. The mean age of the study participants was 68.1 ± 5.8 years (range: 60-86 years for both groups). On subgroup analysis, we found that the severe hyponatremia group had a higher mean age (71.6 ± 6.6 years) as compared to the moderate (66.6 ± 5.1 years) and mild (67.7 ± 5.5 years) ones (p=0.04). Table 1 shows the baseline parameters of the cases and controls. Only sex distribution, hemoglobin, blood glucose, and total leucocyte count showed a statistically significant difference between the groups.

**Table 1:** Characteristics of the study participants P-values in bold indicate statistical significance SD: standard deviation; HTN: hypertension; COPD chronic obstructive pulmonary disease; CKD: chronic kidney disease; UTI: urinary tract infection; AKI: acute kidney injury; DCLD: decompensated chronic liver disease; RBS: random blood sugar; HB: hemoglobin; TLC: total leucocyte count; Na: sodium

Variables	Hyponatremia group (n=100)	Normonatremia group (n=100)	P-value
Males, n (%)	74 (74%)	60 (60%)	0.03
Females, n (%)	26 (26%)	40 (40%)	
Age, years, mean ± SD	68.1 ± 5.8	66.9 ± 5.9	0.15
Comorbidities, n (%)			
Diabetes mellitus	31 (31%)	26 (26%)	0.43
HTN	36 (36%)	30 (30%)	0.36
COPD	10 (10%)	12 (12%)	0.65
CKD	9 (9%)	6 (6%)	0.42
Others	14 (14%)	20 (20%)	0.26
Reason for admission, n (%)			
Cardiovascular disorders	40 (40%)	41 (41%)	0.88
Pneumonia	28 (28%)	37 (37%)	0.17
UTI	8 (8%)	3 (3%)	0.13
Gastroenteritis	6 (6%)	6 (6%)	1
Anemia/AKI	5 (5%)	9 (9%)	0.27
DCLD	5 (5%)	4 (4%)	0.73
Others	12 (12%)	8 (8%)	0.35
Serum potassium (meq/L), mean ± SD	4.6 ± 0.8	4.6 ± 0.5	0.7
RBS (mg/dL), mean ± SD	139.6 ± 57.8	121.1 ± 26.5	0.02
HB (gm/dL), mean ± SD	10.7 ± 2.1	11.9 ± 2.4	0.01
TLC (per mm^3^), mean ± SD	11734 ± 5706	8863 ± 4603	0.004
Serum Na, mean ± SD	129.7 ± 5.1	139 ± 3.4	2.4

Table 2 shows the baseline CGA parameters of the two groups. As evident from this table, all parameters including ADL, TUG, HG, and HMSE had worse scores among the hyponatremia group, but they were significant only for ADL and HMSE. As the severity of hyponatremia increases, all parameters are worsened, while statistical significance is manifested only with respect to changes in TUG and HMSE, as shown in Table 3. This demonstrates that as the severity of hyponatremia increases, there is significantly more decline in cognitive function and increased risk of fall in the elderly as compared to deterioration in other parameters of CGA.

**Table 2:** CGA parameters in the study participants P-values in bold indicate statistical significance CGA: comprehensive geriatric assessment; SD: standard deviation; HMSE: Hindi Mental State Examination; ADL: Barthel Index for Activities of Daily Living; TUG test: Timed Up and Go Test; HG: handgrip

CGA parameter	Hyponatremia group, n=100, mean ± SD	Normonatremia group, n=100, mean ± SD	P-value
ADL	71.6 ± 12.3	76.7 ± 11.5	0.001
TUG	15.4 ± 3.4	15.4 ± 3.3	0.9
HG	9.2 ± 2.4	11.9 ± 3.6	1.6
HMSE	23.4 ± 3.1	24.4 ± 2.4	0.01

**Table 3:** CGA parameters in the participants according to the severity of hyponatremia P-values in bold indicate statistical significance CGA: comprehensive geriatric assessment; SD: standard deviation; HMSE: Hindi Mental State Examination; ADL: Barthel Index for Activities of Daily Living; TUG test: Timed Up and Go Test; HG: handgrip

CGA parameters	Severity of hyponatremia			P-value
	Mild (Na: 130-135 mmol/L) n=66, mean ± SD	Moderate (Na: 125-130 mmol/L) n=20, mean ± SD	Severe (Na: <125 mmol/L) n=14, mean ± SD	
ADL	72.7 ± 10.5	70.4 ± 11.1	66.3 ± 18.4	0.18
TUG	14.6 ± 3.5	16.4 ± 4.2	17.9 ± 3.4	0.003
HG	9.6 ± 2.2	8.6 ± 2.2	8.6 ± 2.9	0.14
HMSE	24.1 ± 2.5	22.6 ± 2.8	21.1 ± 4.0	0.0007

## Discussion

Hyponatremia is one of the most common electrolyte disturbances affecting the elderly population and is seen as a forerunner of many geriatric giants. The present study evaluated the impact of the severity of hyponatremia on commonly used CGA parameters. Our study revealed that even though hyponatremic patients had lower scores for all four CGA parameters, statistical significance was found only in changes related to cognition and ADL. As the severity of hyponatremia increased, TUG and HMSE score changes showed significant differences. The geriatric population has a greater predisposition to hyponatremia because of various associated comorbidities and polypharmacy [13]. Classically, mild hyponatremia is considered asymptomatic, but it has now been shown that it could be associated with fractures, gait disturbances, and impaired cognitive function [4,14-16]. Brinkkoetter et al. conducted an observational study investigating the impact of hyponatremia resolution on the results CGA in 150 patients with age ≥70 years and serum sodium <130 mEq/L. They did the follow-up study and found that improvement was more pronounced in ADL and MMSE after the correction of hyponatremia.

The reason for the decline in cognition due to changes in extracellular sodium levels has been studied. Fujisawa et al. have suggested that chronic hyponatremia directly impairs mitochondrial distribution and decreases the ATP content of neurons, which are known to be induced by excessive glutamate [17]. Therefore, the direct effects of a reduction in extracellular [Na+] on neurons are thought to reinforce neurologic symptoms in conjunction with elevated extracellular glutamate levels. Chung et al. performed a retrospective cohort study and found that hyponatremic patients had a 2.36-fold higher chance of suffering from dementia, including Alzheimer’s disease (AD) and non-AD dementia. Severe hyponatremia patients had a higher risk of suffering from dementia than non-severe hyponatremia patients [adjusted hazard ratio: 4.29 (95% CI: 3.47-5.31) vs. 2.08 (95% CI: 1.83-2.37)]. A dose-response relationship was observed between hyponatremia and dementia [18].

Gosch et al. have demonstrated that geriatric patients with mild-to-moderate hyponatremia revealed a significantly worse outcome in all standardized tests of the geriatric assessment compared to a normonatremic control group [6], which is in line with our findings. Therefore, serum sodium levels should be considered when interpreting common tests of geriatric assessment. Compared to the control group, patients in their study had significantly worse results in all tests of geriatric assessment, including ADL, MMSE, Clock Completion Test, Geriatric Depression Score, Tinetti Mobility Test, the TUG test, and the Mini Nutritional Assessment.

Our study also highlights the importance of monitoring and correcting sodium levels, especially in the geriatric population. The severe hyponatremia group had a higher mean age (71.6 ± 6.6 years) as compared to the moderate (66.6 ± 5.1 years) and mild group (67.7 ± 5.5 years). This is in agreement with many studies demonstrating that the prevalence of severe hyponatremia is significantly higher as the age increases [19]. The study by Brinkkoetter et al. did not detect any significant effects on motor performance [20]. In contrast, Renneboog et al. revealed reversible gait stability impairment in mild-to-moderate hyponatremic patients, which is often found to be responsible for the higher incidence of falls and fractures in the geriatric population [15-16,21-22]. We also found a more significant decline in the TUG score used for fall risk assessment as the severity of hyponatremia increased.

We analyzed the effect of hyponatremia on CGA by comparing the baseline test results of the hyponatremia group with those of the normonatremia group. In line with various studies [6,19], we also observed a significant association of hyponatremia with worse baseline CGA test results. This strongly indicates the true impact of hyponatremia on neurocognitive and motor functions. We found that ADL was the first CGA parameter affected in the mild hyponatremia group (p=0.02). In the moderately hyponatremic group, all the parameters except TUG were significantly lower, whereas all parameters were significantly affected in the severe group. TUG test values were significantly higher in the severe group (17.9 ± 3.4) as compared to the mild (14.6 ± 3.5) and moderate (16.4 ± 4.2s) groups (higher values indicating poor performance). The fact that the statistically significant effects seen with ADL, TUG, and HMSE were not reproducible with the other test suggests that our understanding of the precise impact of hyponatremia is still incomplete.

Our study has some limitations. The baseline CGA values may have been affected by the presence of other underlying diseases. However, we have tried to minimize this by including a normonatremic group as controls during the same study period, who were age-, comorbidity-, and primary diagnosis-matched.

## Conclusions

Based on our findings, hyponatremic patients have poor baseline CGA parameter values, and the severity of hyponatremia correlates with poor motor and cognitive functions. Mild hyponatremia initially presents with lower ADL values. Hyponatremia may present with a myriad of symptoms in the elderly, including delirium, falls, and cognitive disturbance. Serum sodium levels should be strictly monitored, especially in the geriatric population. Hyponatremia correction should be given priority in the elderly. Future studies are warranted to thoroughly analyze the mechanisms and impact of hyponatremia on the geriatric population.
